# Gelatin-methacryloyl hydrogels containing turnip mosaic virus for fabrication of nanostructured materials for tissue engineering

**DOI:** 10.3389/fbioe.2022.907601

**Published:** 2022-09-02

**Authors:** Ivonne González-Gamboa, Edith Velázquez-Lam, Matías José Lobo-Zegers, Ada Itzel Frías-Sánchez, Jorge Alfonso Tavares-Negrete, Andrea Monroy-Borrego, Jorge Luis Menchaca-Arrendondo, Laura Williams, Pablo Lunello, Fernando Ponz, Mario Moisés Alvarez, Grissel Trujillo-de Santiago

**Affiliations:** ^1^ Centro de Biotecnología-FEMSA, Escuela de Ingeniería y Ciencias, Tecnológico de Monterrey, Monterrey, Nuevo León, Mexico; ^2^ Departamento de Bioingeniería, Escuela de Ingeniería y Ciencias, Tecnológico de Monterrey, Monterrey, Nuevo León, Mexico; ^3^ Centro de Biotecnología y Genómica de Plantas, Universidad Politécnica de Madrid—Instituto Nacional de Investigación y Tecnología Agraria y Alimentaria (CBGP, UPM-INIA/CSIC), Madrid, Spain; ^4^ Departamento de Ingeniería Mecatrónica y Eléctrica, Escuela de Ingeniería y Ciencias, Tecnológico de Monterrey, Monterrey, Nuevo León, Mexico; ^5^ Centro de Investigación en Ciencias Físico Matemáticas (CICFIM), Facultad de Ciencias Físico-Matemáticas, Universidad Autónoma de Nuevo León, San Nicolás de los Garza, Mexico; ^6^ Agrenvec SL., Madrid, Spain

**Keywords:** TuMV, GelMA, VNP, nanoscaffold, nanomesh, tissue engineering, bioprinting, biofabrication

## Abstract

Current tissue engineering techniques frequently rely on hydrogels to support cell growth, as these materials strongly mimic the extracellular matrix. However, hydrogels often need *ad hoc* customization to generate specific tissue constructs. One popular strategy for hydrogel functionalization is to add nanoparticles to them. Here, we present a plant viral nanoparticle the turnip mosaic virus (TuMV), as a promising additive for gelatin methacryloyl (GelMA) hydrogels for the engineering of mammalian tissues. TuMV is a flexuous, elongated, tubular protein nanoparticle (700–750 nm long and 12–15 nm wide) and is incapable of infecting mammalian cells. These flexuous nanoparticles spontaneously form entangled nanomeshes in aqueous environments, and we hypothesized that this nanomesh structure could serve as a nanoscaffold for cells. Human fibroblasts loaded into GelMA-TuMV hydrogels exhibited similar metabolic activity to that of cells loaded in pristine GelMA hydrogels. However, cells cultured in GelMA-TuMV formed clusters and assumed an elongated morphology in contrast to the homogeneous and confluent cultures seen on GelMA surfaces, suggesting that the nanoscaffold material *per se* did not favor cell adhesion. We also covalently conjugated TuMV particles with epidermal growth factor (EGF) using a straightforward reaction scheme based on a Staudinger reaction. BJ cells cultured on the functionalized scaffolds increased their confluency by approximately 30% compared to growth with unconjugated EGF. We also provide examples of the use of GelMA-TuMV hydrogels in different biofabrication scenarios, include casting, flow-based-manufacture of filaments, and bioprinting. We envision TuMV as a versatile nanobiomaterial that can be useful for tissue engineering.

## Introduction

Modern tissue engineering relies heavily on hydrogels to support cell growth mainly because these materials closely resemble the extracellular matrix (ECM). Photo-crosslinkable gelatin methacryloyl (GelMA) is one of the most frequently used hydrogels for producing scaffolds and bioinks due to its proven functionality in a wide range of biological applications (Yue et al., 2015) and its suitable rheological properties for extrusion bioprinting (Pedroza-González et al., 2021). The versatile biofunctionality of GelMA arises largely from the presence of gelatin (derived from collagen, its primary source), which contains arginine-glycine-aspartic acid (RGD) motifs that enable cell attachment (Yue et al., 2015; Khashayar et al., 2018). However, GelMA hydrogels often require supplementation with additional chemical and physical cues to turn them into microenvironments that more closely mimic the ECM of specific native tissues (Annabi et al., 2014; Yue et al., 2015; Byambaa et al., 2017; Leijten et al., 2017).

Several strategies have been implemented to engineer suitable hydrogels for tissue engineering. Examples include the addition of micro- and nanoparticles to enhance the mechanical and rheological properties of the hydrogels (Mamaghani et al., 2018; Maharjan et al., 2019; Alcala-Orozco et al., 2020; Bakht et al., 2021; Boularaoui et al., 2021), to provide conductive properties (Shin et al., 2014; Min et al., 2018; Lee et al., 2019), to serve as vehicles for drugs, or to serve as controlled release systems of chemical compounds needed to trigger cell growth proliferation, differentiation, or tissue maturation (Paul et al., 2014; Modaresifar et al., 2017; Samanipour et al., 2020; Tavares et al., 2021). The nanoparticles used in tissue engineering applications are often made of relatively inert materials (i.e., carbon, silica, or metallic nanoparticles) (Hasan et al., 2018).

Functionalization strategies have been devised to engineer nanoparticles to make them more useful for tissue engineering (Elkhoury et al., 2019). For example, nanoparticles have been functionalized with growth factors (Fathi-Achachelouei et al., 2020), peptides (Babitha et al., 2018), cell attachment sites (Lee et al., 2016), antioxidant molecules (Di et al., 2020), and antibacterial compounds (Godoy-Gallardo et al., 2021), among others. Naturally occurring nanoparticles, such as filamentous viruses (i.e., bacteriophages and plant viruses), are now also increasingly recognized as promising nanomaterials and are showing wide use in tissue engineering (Merzlyak et al., 2009; Lauria et al., 2017; Lin et al., 2020; Dickmeis et al., 2021; Jackson et al., 2021; Venkataraman and Hefferon, 2021) and other biomedical applications (Steinmetz and Evans, 2007; Peivandi et al., 2021).

Filamentous viruses are naturally occurring long and hollow protein-based nanoparticles that can be either flexible [like the M13 bacteriophage or the turnip mosaic virus (TuMV)] or rigid [like the tobacco mosaic virus (TMV)] (Zanotti and Grinzato, 2021). The dimensions of filamentous viruses vary between 300 and 900 nm in length and 7 and 15 nm in width (Koudelka et al., 2015). Molecular cloning has enabled the production of viruses as long as 8,000 nm (Jackson et al., 2021). A frequent feature of filamentous viruses is that their capsid consists of a single coat protein (CP) that is repeated thousands of times (Zanotti and Grinzato, 2021). In other words, these viruses are essentially protein-based nanoparticles, which means that they can be conveniently engineered by genetic, chemical, or physical methods, thereby offering a versatile platform for a variety of tissue engineering applications (Yildiz et al., 2012; Song et al., 2015; González-Gamboa et al., 2017; Yuste-Calvo et al., 2019; Velázquez-Lam et al., 2020). However, some issues remain that may limit their applicability.

Filamentous plant viruses and bacteriophages are, in principle, incapable of infecting mammalian cells. (Lauria et al., 2017). Nevertheless, recent evidence now shows that bacteriophage interactions with eukaryotic cells may pose health risks (Podlacha et al., 2021), and more research is needed before plant viruses can be declared safe for use in hydrogels destined for medical implantation. In terms of nanoparticle production, however, the ability to use plants as “green bioreactors” presents some advantages over the traditional culture of bacterial viruses. For instance, plant production does not carry the risk of endotoxin contamination (Magnusdottir et al., 2013) associated with the use of the *Escherichia coli* cultures normally used to propagate bacteriophages. Plant-based production also does not involve the sophisticated equipment and downstream processing required for bacterial-based production (Castiñeiras et al., 2018; Schneier et al., 2020).

The use of plant viral nanoparticles (VNPs) as hydrogel additives for tissue engineering has been explored before. For example, Luckanagul et al. demonstrated the biocompatibility (i.e., low immunogenicity) of hydrogels loaded with TMV and implanted into a mouse model (Luckanagul et al., 2015). Similarly, cultures of a BMSC cell line in a TMV-based HA hydrogel exhibited excellent cell survival and chondrogenesis (Maturavongsadit et al., 2016). Genetically modified potato virus X (PVX) nanoparticles also enhanced cell attachment and promoted an increase in the cell area of bone tissue derived from human mesenchymal stem cells (hMSCs) (Lauria et al., 2017). Recently, Lin et al. demonstrated that the addition of very low quantities of PVX to MSCs cultured on hydrogels enhanced osteogenesis and mineralization (Lin et al., 2020). The use of this plant VNP has also shown advantages in the control of cell orientation, which is relevant for tissue engineering. TMV has been successfully used as a substrate for the culture of C2C12 cells. The production of surfaces with aligned TMV nanoparticles using a controlled fluidic system resulted in an anisotropic nanotopography that promoted the orientation and myogenic differentiation of cultured myoblasts (Zan et al., 2013).

In the present study, we explored the use of TuMV as a nanoadditive for GelMA hydrogels. TuMV is a flexuous filamentous plant virus that belongs to the Potyviridae family. Its specific dimensions are a length of approximately 700–750 nm and a width of 12–15 nm. Its capsid is composed of approximately 2000 repetitions of a single capsid protein, arranged in a helical symmetry, with 8.8 subunits per turn on average (Cuesta et al., 2019; Yuste-Calvo et al., 2019). In a previous report, we provided proof-of-concept data showing that hydrogels coated with TuMV nanoparticles enhance cell attachment and orientation in engineered skeletal-muscle constructs (Frías-Sánchez et al., 2021).

Here, we explored the use of TuMV nanoparticles in their naive version and after functionalization with epidermal growth factor (EGF) in hydrogels destined for culturing fibroblasts (BJ cells) or myoblasts (C2C12 cells). BJ fibroblasts and C2C12 cells are two cell lines frequently used in tissue engineering labs around the world. Therefore, we believe they are convenient models for demonstrating the versatility and wide range of applications provided by the addition of TuMV nanoparticles to tissue engineering applications.

We also report the suitability of this GelMA-TuMV hydrogel for use in different biofabrication techniques (i.e., casting, surface chaotic flows, and 3D bioprinting).

## Results and discussion

**FIGURE 1:**
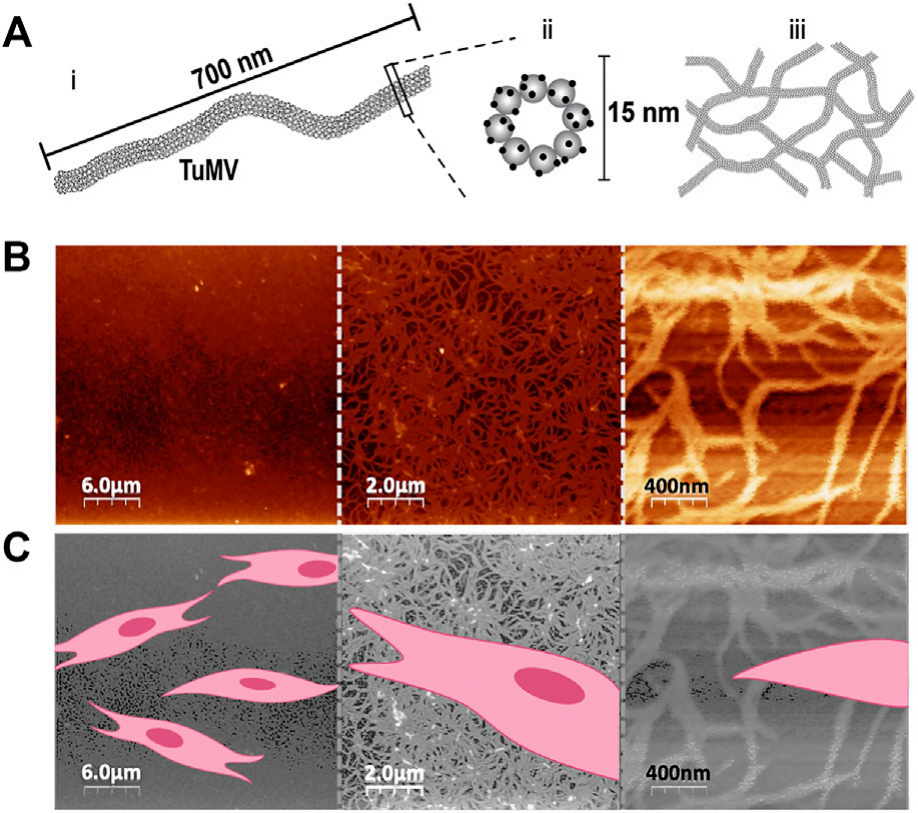
TuMV architecture and size. **(A)** Schematic representation of TuMV size and architecture. The TuMV capsid consists of repeated protein units (shown as gray spheres) that contain lysine amino acid groups that can be chemically functionalized (shown as black dots). Schematic representation of aggregated TuMV forming a nanomesh. **(B)** AFM characterization of TuMV nanomeshes deposited on a silica substrate. **(C)** Schematic representation of the relative size of mammalian cells deposited on a TuMV nanomesh surface.

We report the use of a flexuous plant potyvirus, TuMV (Figure 1A), for functionalization of GelMA hydrogels for tissue engineering purposes. The TuMV nanoparticles were produced and purified from *Brassica juncea* plants following a previously reported protocol (Cuesta et al., 2019). We first assessed the architecture of TuMV by atomic force microscopy (AFM) (Figure 1B) by depositing a drop of a TuMV nanoparticle suspension onto a silica surface and drying at room temperature. The TuMV formed a dense and entangled nanomesh, as revealed by AFM observations (Figure 1B). Similar nanotopographies have been reported for other filamentous viruses, such as the M13 bacteriophage and PVX (Zan et al., 2013; Lee et al., 2016; Lauria et al., 2017).

**FIGURE 2:**
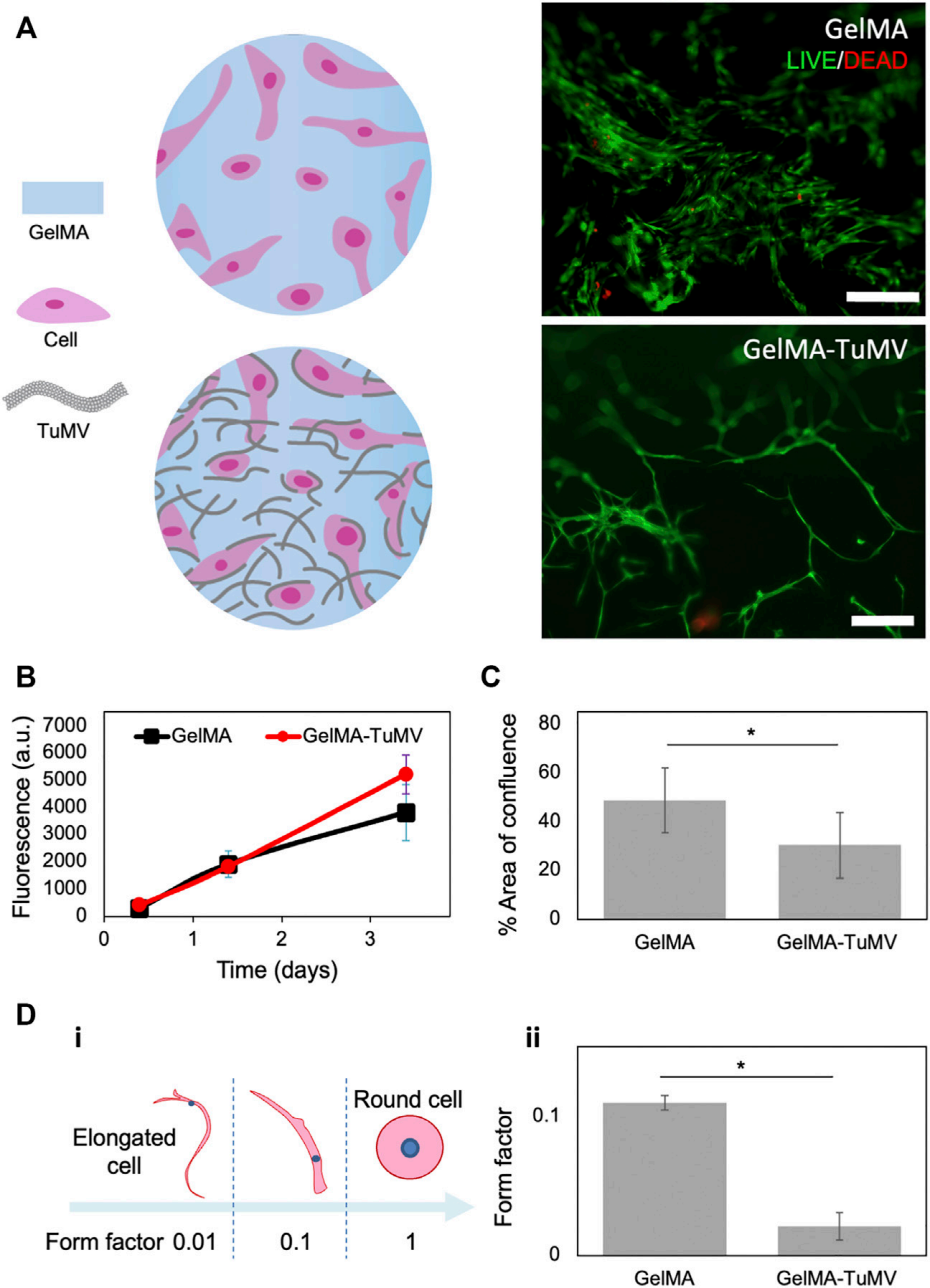
BJ fibroblasts cultured on GelMA hydrogels and on GelMA coated with TuMV. **(A)** LIVE/DEAD^®^ micrographs on day 3. Scale bar 200 μm. **(B)** Metabolic activity measured by PrestoBlue™ assay. **(C)** Evaluation of cell coverage area, and **(D)** cell morphology assessment conducted by image analysis in LIVE/DEAD^®^ micrograph at day 3.

We hypothesized that these TuMV nanomeshes could serve as functional nanoscaffolds for mammalian cell culture. Relative to the typical size of a mammalian cell (Figure 1C), the TuMV nanomeshes present a vast amount of surface area that could foster cell attachment and proliferation. We tested this by culturing human BJ fibroblasts on pristine GelMA hydrogels and on GelMA coated with TuMV nanoparticles (Figure 2A). In these experiments, we monitored the metabolic activity of the cultures every 24 h for 72 h and assessed both the area covered by cells and the cell morphology at the end timepoint.

Cells cultured on both the pristine GelMA and GelMA-TuMV exhibited similar trends in metabolic activity over time, as shown by PrestoBlue™ assays (Figure 2B). LIVE/DEAD^®^ assay micrographs (Figure 2A) indicated very high cell viability (i.e., above 90%) on both materials (i.e., most cells exhibited the green fluorescence signal characteristic of living cells, whereas only a very small number of cells showed red staining indicating dead cells). However, we observed a higher confluence in the cells cultured on pristine GelMA than on the GelMA-TuMV hydrogels (Figure 2C).

Previous studies have reported similar trends. For instance, Lee et al. (2016) observed high viability of pre-osteoblast cultures in alginate-based bioinks containing M13 bacteriophages. They compared the performance of wildtype phages and phages genetically modified to display RGD peptides on their surfaces (RGD-phage) in cell culture experiments. The authors found that the cell viability was high for the alginate hydrogels enriched with both wildtype and RGD-functionalized phages. However, the proliferation rates observed with the wildtype phage-loaded bioinks were lower than those observed for the RGD-alginate bioink and alginate containing the RGD phages (Lee et al., 2016). In our experiments, the effect of the presence of TuMV on cell morphology was clear (Figure 2D). Cells formed aggregates and developed a significantly more elongated morphology when cultured on GelMA-TuMV hydrogels than on pristine GelMA. We conducted image analysis to quantitatively compare the morphology of cells cultured on both substrates. Cell elongation was analyzed by calculation of the form factor, an indicator frequently used to characterize cell roundness. Form factors of cells with a more rounded morphology tend to 1, while those describing very elongated cells tend to 0.01 (Figure 2Di). Fibroblasts cultured on GelMA-TuMV exhibited significantly lower form factors (0.02 ± 0.01) than those cultured on pristine GelMA (0.11 ± 0.02). In other words, cells developed longer and thinner morphologies when cultured in the presence of TuMV than when cultured without TuMV (Figure 2Dii).

This finding is consistent with previous reports that nanotopographic features provided by filamentous viruses influenced cell morphology. For instance, Yoo et al. found that human fibroblasts developed more elongated morphologies when cultured on patterned surfaces containing M13-phage functionalized with RGD peptides than on substrates containing RGD peptides (but free of VNPs) (Yoo et al., 2011). The same authors also tested phages functionalized with RGE peptides as a non-cell-adhesive control. As expected, cell attachment and elongation were lower in cultures containing RGE peptides than in the RGD-phage counterparts, but the cells still exhibited a more elongated morphology when cultured on RGE-phage substrates than on substrates free of VNPs. In another study, Merzlyak et al. (2009) observed that neural progenitor cells (NPCs) cultured on M13-phage casted surfaces tended to form cell clusters, whereas NPCs cultured on laminin-coated surfaces spread homogenously. This differential behavior was attributed to a poor affinity between the cells and the phage-coated surface. The same authors also demonstrated that functionalizing the capsid coat protein (CP) of the phage with protein adhesive sites (e.g., RGD and a laminin-like peptide) enhanced cell spreading on the phage-coated surfaces (Merzlyak et al., 2009).

Our results show that the addition of non-functionalized TuMV to GelMA hydrogels induces clear differences in the resulting morphology and confluence of the cultured cells. However, these differences do not translate into either a more effective attachment or better proliferation. GelMA hydrogels naturally contain RGD domains that are preserved from collagen. However, when the GelMA surface is covered with the TuMV nanomesh (a material that does not contain specific cell-adhesion sites), cell attachment is partially obstructed. The lengthened morphology may be a response of the cells when they sense the dense nano-topographical features when growing on a substrate with relatively poor affinity for cell adhesion.

Overall, these results suggest that cells respond to the nanoscaffold provided by the TuMV nanomesh, and that the addition of TuMV to GelMA has a clear effect on the morphology of fibroblasts by triggering unusual elongation. This morphological change probably arises due to TuMV interference with the proper attachment of the cells to GelMA surfaces but does not negatively affect cell viability.

### Engineering GelMA-TuMV hydrogels with epidermal growth factor

**FIGURE 3:**
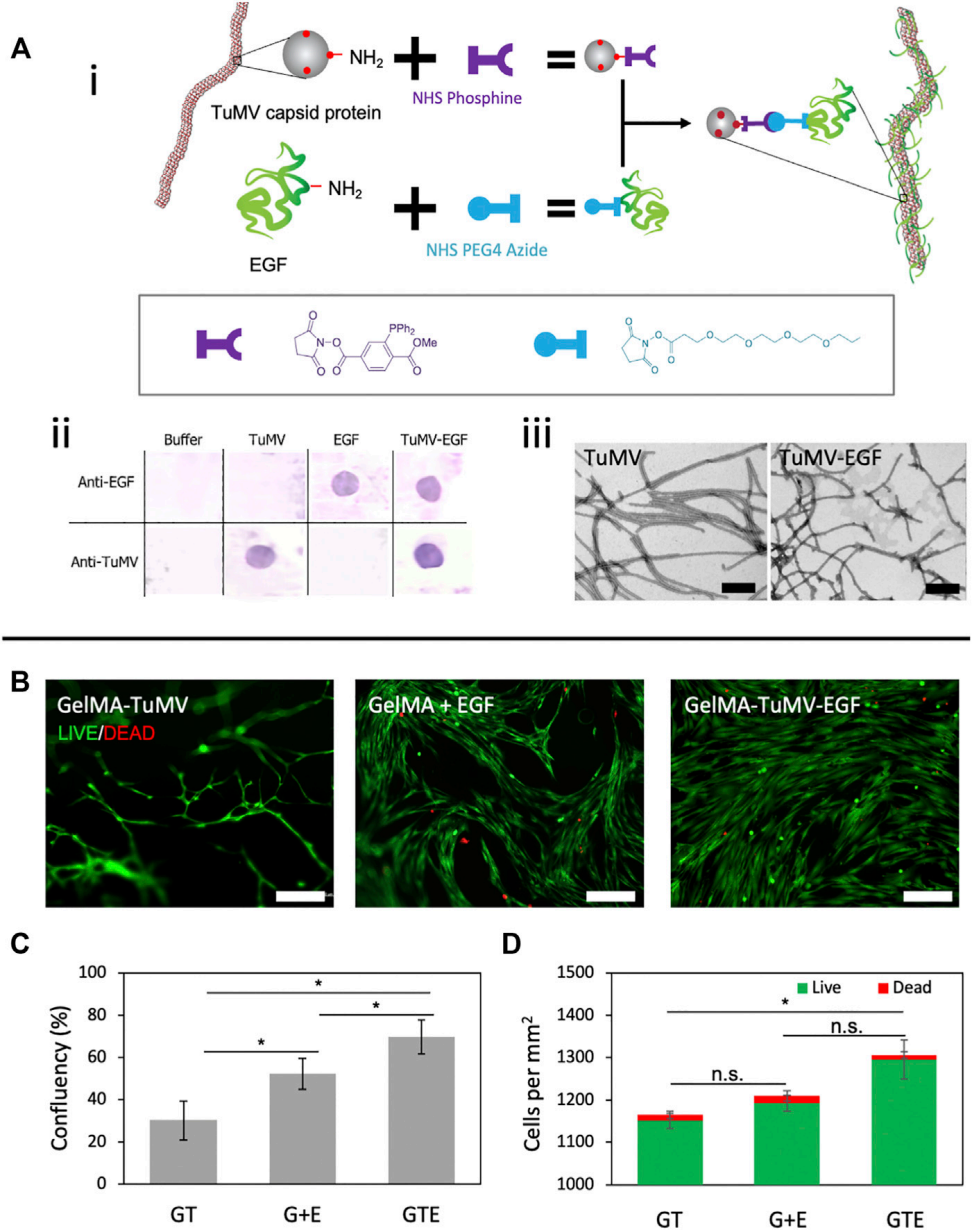
TuMV-EGF bioconjugation and its effect on cell proliferation. **(A)** Bioconjugation strategy to tether EGF onto TuMV. (i) Schematic representation of the conjugation via a Staudinger reaction between phosphine and azide. (ii) Dot-Blot immunoassay revealing the successful conjugation of EGF on TuMV surface. (iii) Morphology of TuMV and TuMV-EGF nanomeshes visualized by TEM. Scale bars 200 nm. **(B)** LIVE/DEAD^®^ assay of BJ fibroblasts cultured on GelMA coated with TuMV [GelMA-TuMV (GT)], GelMA with added EGF in the cell culture media [GelMA + EGF (G + E)], and GelMA coated with bioconjugated TuMV (GelMA-TuMV-EGF [GTE]) for 72 h. Scale bars = 200 μm. **(C)** Confluence, and **(D)** counts of live cells and dead cells evaluated by image analysis from LIVE/DEAD^®^ micrographs (*n* = 4).

We also functionalized the TuMV capsid with epidermal growth factor (EGF). Our aim in this set of experiments was to demonstrate the tailoring of the surface of TuMV particles to enhance their functionality for tissue engineering purposes. EGF has been shown to stimulate or mediate cell attachment (Thorne et al., 1987; Zhang et al., 1996; Pansani et al., 2019) and proliferation (Thönes et al., 2019; Hu et al., 2021). We exploited the readily available lysine residues projected on the TuMV surface (Cuesta et al., 2019) to graft the EGF molecules (Figure 3A).

We first grafted phosphine and azide groups to the N-terminal domains of the lysine residues exposed at the TuMV surface and EGF, respectively, using an NHS-ester reaction (Figure 3Ai). We then conducted a Staudinger reaction (Saxon and Bertozzi, 2000), as reported previously by our group (Yuste-Calvo et al., 2019), to functionalize the TuMV nanoparticle. This strategy involves the reaction between phosphine and azide groups that occurs under physiological conditions. We confirmed the successful TuMV-EGF conjugation using Dot-Blot assays. As expected, samples containing EGF (either conjugated to TuMV or as free molecules) reacted with anti-EGF antibodies. Samples containing TuMV (conjugated or pristine) reacted with anti-TuMV antibodies (Figure 3Aii). We did not conduct an experimental determination of the degree of EGF functionalization in our TuMV meshes. However, we can provide a good approximation of the EGF content in our TuMV-EGF based on stoichiometric arguments, because the entire train of the reaction is conducted aiming for a high degree of conjugation. First, EGF is reacted with a 3-fold excess of HHS-azide. Similarly, the ligation of TuMV to the phosphine reagent is conducted with a 3-fold excess of NHS-phosphine.

Since the TuMV capsid consists entirely of the capsid protein (CP), the molecular weight of the CP (i.e., 33 kDa) is assumed to be the molecular weight of TuMV and is used to convert mass to molar quantities. Finally, the reaction between the azide-labeled EGF and the phosphine-labeled TuMV is conducted in a 100% excess of EGF-azide. In addition, both the azide-NHS and the phosphine-NHS react with lysine residues, and more than one lysine residue is available per molecule in both the CP and the EGF molecules. The CP of TuMV contains 22 lysine residues (Yuste-Calvo et al., 2019). EGF possesses two lysine residues; one of them is available in the proximity of the C-terminal.

Considering all these stoichiometric arguments, a high degree of functionalization of TuMV nanoparticles with EGF should be achieved (i.e., ∼0.18 µg of EGF per µg of TuMV if at least one EGF molecule reacts per CP unit). The Staudinger reaction (the reaction between the azide compound and the phosphine compound) also normally has high conversion yields (i.e., above 90%) (Sundhoro et al., 2017; Bednarek et al., 2020).

Further evaluation of the morphology of TuMV nano-meshes before and after EGF conjugation using transmission electron microscopy (TEM) (Figure 3Aiii) revealed no major differences in size (width/length) or morphology between the conjugated and pristine TuMV. These morphological observations are consistent with previous work reporting the covalent functionalization of TuMV with different types of molecules (Yuste-Calvo et al., 2019; Velázquez-Lam et al., 2020).

We assessed the performance of the TuMV-EGF as a cell nanoscaffold using human fibroblasts. For this, BJ cells were cultured for 72 h in three types of hydrogels: GelMA coated with EGF-conjugated TuMVs (GelMA-TuMV-EGF), GelMA coated with pristine TuMV (GelMA-TuMV), and GelMA with EGF added to the culture medium (GelMA + EGF). We observed significantly higher cell-confluence values (i.e., the area covered by cells) in GelMA-TuMV-EGF samples than in GelMA-TuMV or GelMA + EGF cultures (Figures 3B,C).

Consistently, the number of cells per unit of area was higher in GelMA-TuMV-EGF samples than in their counterparts (Figure 3D). LIVE/DEAD assays (Figure 3B) revealed that all the tested conditions resulted in cell viabilities higher than 98% (Figure 3D). Fibroblast cultures on TuMV-EGF substrates showed a more homogeneous coverage and cell morphology than cultures growing on GelMA and GelMA with unconjugated EGF. These results suggest that the multimeric presentation of EGF on the surface of TuMV (over 2000 theoretical sites of conjugation per nanoparticle) may effectively enhance the surface area that mammalian cells sense for attachment or may stimulate fibroblast proliferation (Yuste-Calvo et al., 2019).

### GelMA-TuMV hydrogels used in biofabrication

**FIGURE 4:**
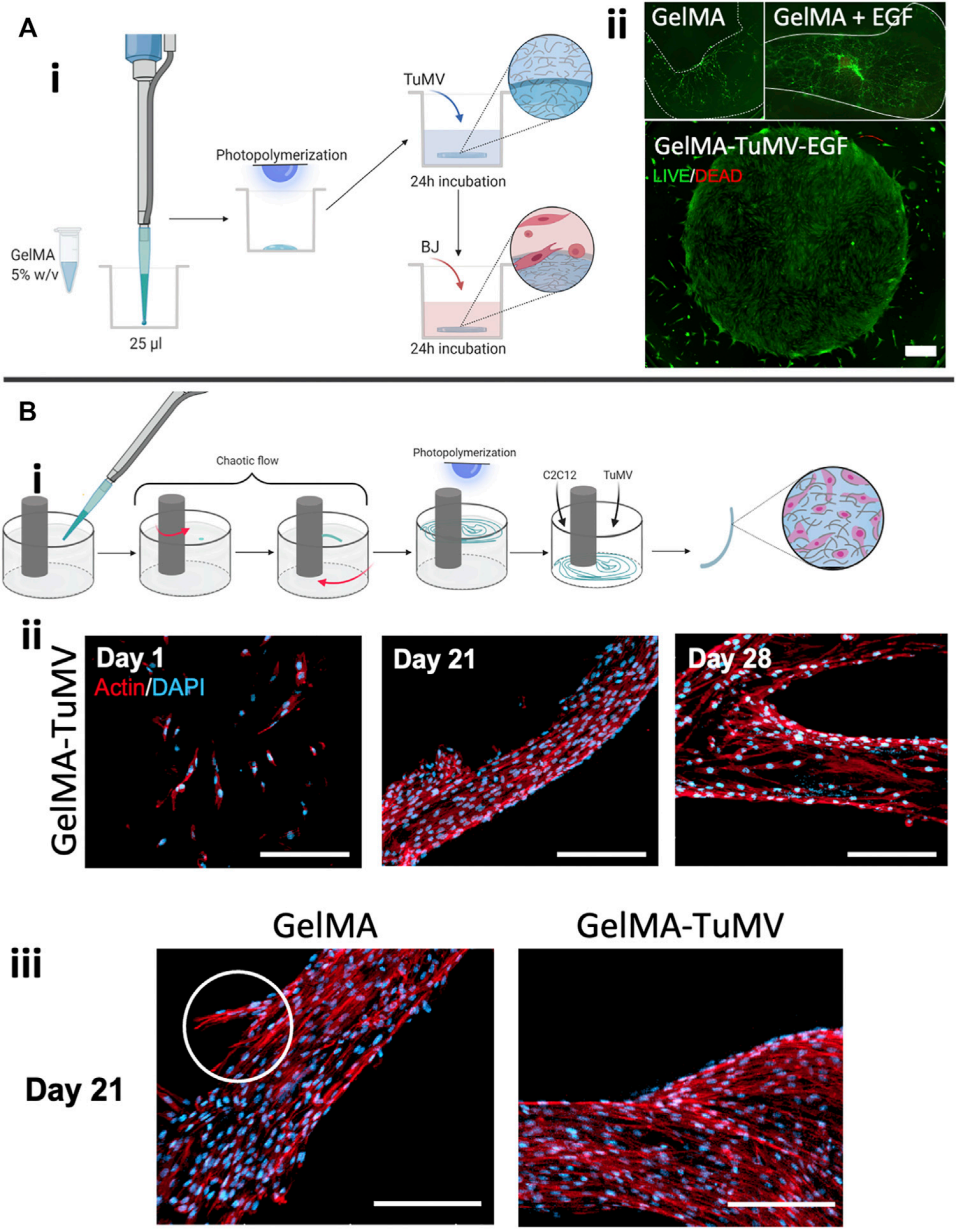
GelMA-TuMV hydrogel constructs produced by different biofabrication techniques. **(A)** Disk-shaped constructs produced by casting in ultra-low attachment (ULA) plates. (i) Schematic representation of the fabrication process. (ii) LIVE/DEAD^®^ assay of BJ fibroblasts cultured on the disks after 7 days of culture. Scale bar = 500 μm. **(B)** Fiber-shaped constructs produced by surface chaotic flows. (i) Schematic representation of the fabrication process, (ii) Actin/DAPI staining of C2C12 myoblasts cultured on the TuMV coated GelMA fibers over time. (iii) Actin/DAPI staining of C2C12 myoblasts cultured on pristine GelMA fibers and GelMA-TuMV fibers after 21 days of culture. Scale bars = 100 μm.

We also evaluated the use of GelMA-TuMV to fabricate constructs using different biofabrication strategies. First, we explored the manufacturing of GelMA disks coated with TuMV nanoparticles and then seeded them with BJ fibroblasts on their surfaces. Briefly, GelMA disks (1.6 mm in diameter) were produced by simply adding a drop of GelMA pregel to an ultra-low-attachment (ULA) well and subsequently photocrosslinking using UV light. The GelMA disks were then coated with a suspension of TuMV (10 μg/ml), incubated overnight at 4°C, and seeded with BJ cells (Figure 4Ai). The observed cell confluence was significantly higher and more homogeneous on GelMA-TuMV-EGF disks than on GelMA and GelMA + EGF disks (Figure 4Aii). Interestingly, the disks coated with TuMV conserved their shape over the culture time, while those without TuMV deformed and folded. These results suggest that GelMA-TuMV-EGF provides a hierarchical scaffold (at the micrometer and nanometer scales) that provides functional molecules locally (EGF) for cell attachment, proliferation, and spreading, thereby fostering the generation of confluent microtissues in a few days (7 days) (Figure 4Aii). We envision that this simple strategy for the fabrication of densely populated hydrogel disks could enable the facile fabrication of thick multilayered constructs simply by stacking multiple disks.

We also explored the fabrication of thin GelMA filaments coated with TuMV nanoparticles for the potential development of skeletal-muscle-like tissues using murine myoblasts (C2C12 cells). We produced long (i.e., approximately 5 cm long) and thin (i.e., 100–300 μm thick) filaments of GelMA using surface chaotic flows (Figure 4Bi). For this, we used an in-house constructed device (the mini Journal Bearing), as reported previously by our group (Frías-Sánchez et al., 2021) (Figures 4Bii,iii).

Similar to the GelMA disks, these filaments were coated with a TuMV suspension (10 μg/ml) and then surface seeded with C2C12 cells. Figures 4Bii,iii show that GelMA-TuMV filaments successfully supported the attachment and proliferation of C2C12 cells for extended culture periods (up to 28 days). We observed a confluent cell population and a dominant orientation on the surface of the filaments, particularly at its narrower sections (Figure 4Bii). Similar cell behaviors were observed in both GelMA and GelMA-TuMV filaments. However, we consistently observed a stronger cell attachment to GelMA-TuMV surfaces than to pristine GelMA filaments. We also observed a higher occurrence of cell detachment over time in GelMA filaments than in GelMA-TuMV filaments (Figure 4Biii).

**FIGURE 5:**
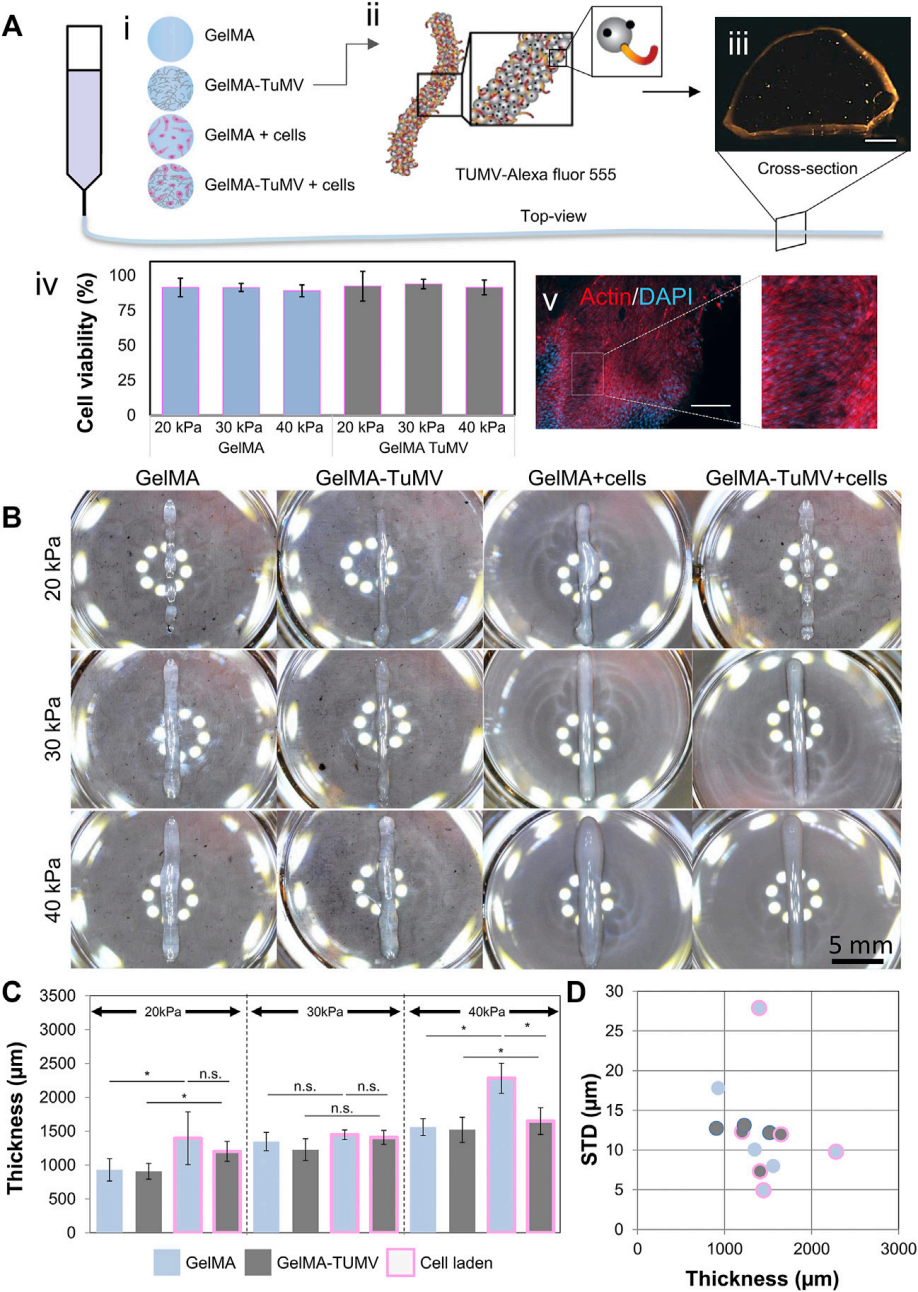
GelMA-TuMV bioprinting. **(A)** Hydrogels tested for extrusion bioprinting. (i) Schematic representation showing the composition of the hydrogels. (ii) Schematic representation of the functionalization of TuMV with Alexa Fluor 555 and (iii) a micrograph of the cross section of a filament printed with GelMA-TuMV-Alexa Fluor 555. Scale bar = 200 μm. (iv) Cell viability immediately after bioprinting using GelMA or GelMA-TuMV inks at different pressures, and (v) Actin/DAPI staining of filaments printed with GelMA-TuMV loaded with C2C12 cells after 21 days of culture. Scale bar = 200 μm. **(B)** Photographs of lines printed using different hydrogels. Scale bar = 5 mm. **(C)** Analysis of the thickness of the printed lines, and **(D)** the standard deviation of the thickness of fibers, as calculated by image analysis.

We also explored the use of our hydrogels in 3D printing experiments conducted with a commercial extrusion bioprinter at three different pressure settings (i.e., 20, 30, and 40 KPa). In this set of experiments, TuMV was dispersed in the bulk phase of GelMA pre-gels (i.e., not deposited on the surface) to produce printing inks that were later evaluated in terms of printability. We tested four materials: pristine GelMA, GelMA loaded with TuMV (GelMA-TuMV), GelMA loaded with cells (GelMA + cells), and GelMA loaded with TuMV and cells (GelMA-TuMV + cells) (Figure 5Ai).

We first confirmed that the TuMV nanoparticles were homogeneously dispersed in the GelMA. To do this, we conjugated TuMV nanoparticles with Alexa Fluor 555 to render them visible by fluorescence microscopy (Figure 5Aii). Once the dye was conjugated, we suspended 10 μg/ml of the VNPs on GelMA and printed lines using a BioX (CELLINK, Sweden) bioprinter. Figure 5Aiii shows a cross section of the printed filament evidencing the presence of the TuMV nanoparticles throughout the sample (the orange signal was well distributed in the filament slice; Figure 5Aiii). As further proof-of-concept evidence, we bioprinted lines using GelMA-TuMV bioinks loaded with C2C12 cells. The bioinks were suitable for extrusion bioprinting. Figure 5Aiv shows post printing viabilities for different pressure setting as measured in LIVE/DEAD assays. In all cases, average cell viabilities ranged between 82 and 90% and we did not observe a significant effect of different pressures on cell viability. In addition, bioprinted filaments sustained cell growth for long periods (21 days; Figure 5Av).

The printability of the bioinks was quantitatively assessed by evaluating the morphology of lines printed with them at three different extrusion pressures (20, 30, and 40 kPa). Ideally, the printed lines should be straight, be as thin as the diameter of the printing nozzle (410 μm), and exhibit a uniform thickness along their length. The properties of the inks should also enable reproducible printings. We evaluated the thickness of the printed lines (12 arbitrary sections along the same line) using image analysis (Figures 5B–D).

The rheological and mechanical properties of inks are very relevant in bioprinting and greatly determine printability (Schwab et al., 2020). Indeed, the addition of nanoparticles may affect the properties of the inks. However, in the printing experiments reported here, we added low concentrations of nanoparticles to our GelMA ink. Therefore, the rheological and mechanical properties of GelMA-TuMV inks were expected to be similar to those exhibited by pristine GelMA hydrogels. To corroborate this, we evaluated mechanical and the rheological properties of our GelMA and GelMA-TuMV inks. As expected, the compressive modulus of GelMA and GelMA-TuMV hydrogels was similar (Supplementary Figure S1). The curves of viscosity versus shear rate curves of pristine GelMA and GelMA-TuMV inks were also similar (Supplementary Figure S2A). Consistently, the curves of viscosity at different temperatures were practically indistinguishable between GelMA and GelMA-TuMV inks (Supplementary Figures 2B,C).

We also present an assessment of filament uniformity by characterizing the variations in thickness along filaments printed with GelMA, GelMA-TuMV, and GelMA with cells (Schwab et al., 2020). In general, the addition of TuMV particles did not negatively affect the printability of GelMA-based inks (without cells) at any of the tested pressure conditions. Our printing experiments suggest that the shape fidelity was statistically similar in both GelMA-TuMV and GelMA. The thicknesses of the filaments printed with GelMA and GelMA-TuMV were statistically similar across a wide range of printing pressures.

Our results also suggest that the addition of TuMV particles to bioinks (i.e., inks containing living cells) represents a simple strategy to improve printability. Cells generally have an impact on the rheological properties of inks (Schwartz et al., 2020); the printability of inks may be negatively affected by the addition of cells. The addition of TuMV particles to GelMA bioinks decreased the thickness of the printed lines. The average thickness was smaller in lines printed with GelMA bioinks with added TuMV than without added TuMV (Figures 5B,C). The lines also exhibited a higher homogeneity in thickness when printed with GelMA-TuMV bioinks than when printed with bioinks without TuMV; the standard deviation of the thicknesses was lower for GelMA-TuMV lines than for GelMA lines (Figure 5D). Interestingly, the average thickness of cell-laden GelMA lines printed at the highest pressure value (i.e., 40 kPa) was significantly greater (>1,200 μm) with cells (and no TuMV added) than without cells (∼900 μm). We attribute the difference in thickness in that printed line mainly to the lower crosslinking ability of GelMA inks because of the presence of cells. In general, a slightly greater thickness is observed in cell-laden lines than in lines not containing cells. This effect is enhanced by the high pressure, which causes a higher flow rate and therefore higher mass deposition per unit time and, consequently, greater thickness.

Remarkably, in these bioprinting experiments at high pressures, the average thickness is much lower for GelMA-TuMV filaments than for GelMA filaments. This suggests that the addition of TuMV to our bioinks has a positive effect on shape fidelity at high pressures.

## Conclusion

We have introduced the use of flexuous plant viral nanomeshes (i.e., TuMV particles) that can serve as a functional additive for GelMA hydrogels. The TuMV acts as a nano-topographical scaffold for cells and provides a protein framework for further biological-specific functionalization. We illustrate a strategy to develop tailored hydrogels for the controlled release of EGF. The presence of TuMV-EGF on the surface of hydrogels promotes cell attachment and accelerated proliferation of fibroblasts. This results in the development of tissue constructs with high confluence.

We also explored the use of TuMV particles in three different biofabrication scenarios. First, we illustrated a simple method for the fabrication of disks of GelMA coated with TuMV and seeded with BJ fibroblasts. We then demonstrated the use of surface flows to produce long and thin filaments of GelMA surface coated with TuMV particles to produce highly aligned microfibers of muscle-like tissue. GelMA bioinks containing added TuMV particles also exhibited satisfactory printability in bioprinting experiments using a commercial bioprinter.

These engineered hydrogels functionalized with TuMV particles have the potential for use in medical scenarios, such as regenerative treatments for burn injuries, post-surgery incisions, and chronic wounds. Future work will include the customization of TuMV-GelMA inks for different applications through exploitation of the bioconjugation of the TuMV capsid with various bioactive molecules or peptides.

In the context of tissue engineering applications that involve implantation, one concern is the fate of TuMV nanoparticles in the human body. Plant viruses are, in principle, incapable of infecting mammalian cells (Lauria et al., 2017; Balke and Zeltins, 2020). Due to their protein nature, they are expected to eventually undergo enzymatic degradation under physiological conditions (Nkanga et al., 2022). However, to our knowledge, no experiments have assessed the fate of implanted hydrogels containing TuMV. In a recent study, Velázquez-Lam injected healthy and tumor-induced mice intravenously with TuMV particles marked with Alexa and traced the particles for several days (Velázquez-Lam, 2021). The TuMV concentration in blood dropped linearly, suggesting full blood clearance in less than a week. The accumulation in different tissues was also measured. By the fourth day of intravenous injection, the highest accumulation was observed in the liver, distinctly followed by the tumors, lymph nodes, kidneys, and spleen.

The literature also has no antecedents of the use of TuMV particles in clinical studies. However, several plant viruses have been tested in human and veterinary studies in the context of the development of vaccine platforms (Balke and Zeltins, 2020). Overall, the results suggest that plant virus-based vaccines could be introduced into clinical and veterinary practice in the near future (Folia Biotech Inc, 2000; Chichester et al., 2018). More research is needed before plant viruses can be declared safe for use in hydrogels destined for medical implantation, particularly as several plant viruses have proven to be potent adjuvants (Folia Biotech Inc, 2000; Sánchez et al., 2013; Chichester et al., 2018; Nkanga et al., 2022) and may trigger clinically relevant immune responses if implanted.

## Materials and methods

### GelMA synthesis

In all the experiments reported here, Gelatin methacryloyl (GelMA) medium i.e., gelatin methacryloyl with a medium degree of methacrylation (47–62% methacrylation) as determined by NMR techniques (Sánchez-Rodríguez, 2019). GelMA medium was synthesized following the protocols reported elsewhere, with a few modifications (Bolívar-Monsalve et al., 2021). Briefly, we dissolved porcine gelatin type A (Sigma-Aldrich, United States) in Dulbecco’s phosphate-buffered saline (DPBS) (Invitrogen Life Technologies, United States) at 10% (w/v) under constant stirring at 400 rpm at 50°C for 1 h. Then, 10% (v/v) of anhydride methacrylate (Sigma-Aldrich, United States) was added dropwise to the gelatin solution using a syringe pump at a flow rate of 0.5 ml/min and magnetic stirring for 1 h. The reaction was stopped by adding DPBS to the solution at a 1:5 ratio.

Subsequently, the solution was dialyzed against distilled water at 40°C for 7 days using dialysis tubing and constant stirring. The dialysis water was continuously refreshed using a peristaltic pump. Finally, the GelMA was frozen at −80°C, freeze-dried for 5 days, and stored at 4°C until its use.

### TuMV production, conjugation, and characterization

#### Production and purification of TuMV nanoparticles

Turnip mosaic virus (TuMV, isolate UK 1) was propagated in plants of Indian mustard (*Brassica juncea*) and harvested 30 days post-inoculation. TuMV nanoparticles were purified from 150 g of plant material, as described by Sánchez and Ponz (2018). Briefly, plant tissue was finely ground in 0.5 M potassium phosphate, pH 7.5, 1:2 (w/v), in an electrical tissue grinder, at 4°C. The resulting suspension was extracted with one volume of chloroform at 4°C. The phases were separated by centrifugation, and the aqueous phase was filtered through Miracloth (MerckTM, Germany). The TuMV nanoparticles in the filtrate were precipitated with 6% PEG 6,000 (w/v) and 4% NaCl (w/v) and allowed to settle for 90 min at 4°C. The particles were recovered by centrifugation at 12,000 g for 10 min. The pellet was resuspended overnight in 0.5 M potassium phosphate, pH 7.5, and 10 mM ethylenediaminetetraacetic acid (EDTA). The suspension was clarified by centrifugation (10 min at 9,000 g), and the TuMV nanoparticles were pelleted (2 h at 80,000 g). The pellet was resuspended in 0.25 M potassium phosphate, pH 7.5, and 10 mM EDTA, and CsCl was added to a final density of 1.27 g/cm^3^. The resulting suspension was centrifuged at 150,000 g for 18 h at 4°C.

A visible band in the gradient containing the TuMV particles was recovered by punching the tube with a syringe and needle. This band was diluted in a solution of 0.25 M potassium phosphate and 10 mM EDTA (pH 7.5) and then centrifuged at 80,000 g for 2 h. Finally, the generated pellet was resuspended in a glycerol solution (i.e., 50% glycerol (v/v), 5 mM Tris, and 5 mM EDTA at pH 7.5) at a final concentration of 1 mg/ml and stored at −20°C until further use. The TuMV nanoparticle concentration was determined spectrophotometrically using an absorption coefficient of 2.65 (A0.1%, 1 cm at 260 nm).

#### EGF conjugation to TuMV

TuMV nanoparticles were centrifuged (50 min, 60,000 g, 4°C) and then resuspended in 10 mM HEPES (Sigma-Aldrich, United States) at pH 7.5, to a final concentration of 1 mg/ml. The ligation started with the addition of a 3-fold molar excess of NHS-phosphine (Thermo Scientific Pierce, United States) to a 1 mg/ml suspension of TuMV nanoparticles, followed by overnight incubation in the dark at 4°C. The reaction mix was centrifuged (50 min, 60,000 g, 4°C) and the excess NHS-phosphine was eliminated in the supernatant. The pellet was washed with 10 mM HEPES and then resuspended to a final concentration of 1 mg/mL. A linker NHS-azide (Thermo Scientific Pierce, United States) was added to a plant-made EGF (Agrenvec, Spain) in a 3-fold molar excess, followed by an overnight incubation in the dark at 4°C. The reaction mix was filtered through an Amicon (Merck, United States) centrifuge filter, and the excess NHS-azide was eliminated in the filtrate. After the reaction between TuMV and the phosphine reagent, we added a 1-fold molar excess of the modified EGF. The reaction was incubated overnight in the dark at 4°C. The excess modified EGF was removed by centrifugation (50 min, 60,000 g, 4°C). Samples were stored at 4°C for further use.

#### Preparation of fluorescent TuMV nanoparticles

TuMV nanoparticles were tagged with Alexa™ FluorTM 555 NHS Ester (Life Technologies, United States) following the manufacturer’s protocol with some modifications. The suspension buffer was changed for HEPES 20 mM pH 7.5 by ultracentrifugation (50 min at 50,000 g). The succinimidyl esters of AlexaTM FluorTM were covalently attached to primary amines at the capsid surface. To do this, a three-fold excess of the Alexa Fluor reagent was added to a 30 µM TuMV suspension. This mixture was stirred and incubated in the dark for 2 h at room temperature. Unbonded Alexa was removed by ultracentrifugation (50 min at 50,000 g) and the pellet was resuspended with HEPES 20 mM pH 7.5 at 30 μM of TuMV.

#### Atomic force microscopy

For the characterization with AFM, silica wafers (DoñaChip, Mexico) were cut into ∼1.5 cm^2^ squares. The surfaces were washed with 96% ethanol before use, coated with 5 μL of 0.02 mg/ml TuMV suspension, and dried overnight in a sterile environment. The TuMV coverage was analyzed by Ntegra NT-MDT spectrum Instruments AFM used in semi-contact mode with silicon probes (Tap190AI-G^®^, United States) with a resonance frequency of 190 kHz, a spring constant of approximately 48 N m^−1^, and a scan rate of 1.0 Hz.

#### Transmission electron microscopy

Electron microscopy grids (nickel formvar-coated, 400 mesh and copper/carbon-coated, 400 mesh; Cientifica Senna™, Spain) were placed over a drop of TuMV nanoparticle suspension, incubated for 10 min at room temperature, and then washed with a 50 mM borate buffer (pH 8.1). The microscopy grids were stained with 2% uranyl acetate, and the samples were examined with a transmission electron microscope (JEM 1400, Spain).

#### Dot-blot assays

EGF conjugation to TuMV was assessed by adding 10 μL of sample (pristine TuMV and TuMV conjugated with EGF) to a dry polyvinylidene difluoride (PVDF) membrane (Trans-Blot Transfer Medium, Bio-Rad) and allowing the sample to absorb completely. The membrane was placed overnight in a blocking solution [2% non-fat dry milk in PBS containing 0.05% Tween 20 (PBST)] at 4°C. The membrane was then washed three times by 5 min immersions in PBST. The membrane was then immersed in an anti-TuMV antibody (Agdia, United States) solution for 1 h at room temperature and washed three times as previously described. The membrane was immersed in an anti-EGF antibody (Agrenvec, Spain) solution for 1 h at room temperature and washed as previously described. Subsequently, the membrane was immersed in an anti-mouse AP (Agdia, United States) antibody (Agrenvec, Spain) solution for 1 h at room temperature and washed as previously described. For visualization, the membrane was incubated in an NBT-BCIP solution at room temperature until the samples were revealed.

### Cell culture experiments

#### Cell lines

Human BJ fibroblasts (CRL-2522) and C2C12 mouse myoblasts (CRL-1772TM) were obtained from ATCC^Ⓡ^. Culture media and reagents were purchased from (Invitrogen™, United States), unless indicated otherwise. BJ cells were cultured in Eagle’s minimum essential medium (EMEM). C2C12 cells were cultured in Dulbecco’s Modified Eagle’s Medium (DMEM). The culture media for both cell lines were supplemented with 10% (v/v) fetal bovine serum (FBS) and 1% (v/v) penicillin-streptomycin. Cells were grown at 37°C in a 5% CO_2_ atmosphere.

#### Cell culture experiments on well surfaces coated with GelMA

The wells of 96-well plates were coated with 5% GelMA. To do this, a solution of 5% w/v GelMA prepared in DPBS was incubated in a water bath at 37°C until complete dissolution. The lithium phenyl-2,4,6-trimethylbenzoylphosphinate (LAP; CELLINK, Gothenburg, Sweden) photoinitiator was then dissolved at a final concentration of 0.067% (w/v). The GelMA solutions were sterilized by syringe filtration through 0.22 μm polyethersulfone (PES) membranes. A 25 μL volume of GelMA was deposited in each well and crosslinked by UV light exposure at 365 nm for 30 s.

TuMV, EGF, or TuMV-EGF solutions (10 μg/ml) were then added to the pre-coated wells and allowed to attach for 3 h at 4°C. DPBS washes were performed to remove non-attached reagents. BJ fibroblasts (4 × 10^4^ per well) were then seeded and cultured for 72 h at 37°C in a 5% CO_2_ atmosphere.

#### Assessment of metabolic activity

The metabolic activity of the cell cultures was determined using PrestoBlue™ (Invitrogen) assays. We added 10% (v/v) PrestoBlue™ reagent (with respect to the culture medium) to the samples contained in 96-well plates, followed by incubation at 37°C for 1 h. The fluorescence intensity was measured with a microplate reader (Synergy HTX Multi-mode, BioTek, United States) at excitation/emission wavelengths of 530/570 nm. The fluorescence intensities were normalized with respect to the first reading at 12 h.

#### LIVE/DEAD^®^ staining

The LIVE/DEAD^®^ assays (Invitrogen) were conducted according to the manufacturer’s instructions. Briefly, the culture medium was removed from cells or printed constructs and replaced by HEPES (2-[4-(2-hydroxyethyl) piperazin-1-yl]ethanesulfonic acid). The LIVE/DEAD^®^ reagent was then added to the samples and incubated for 20 min at room temperature, followed by three washes with PBS. Fluorescence images were obtained using an Axio Observer. Z1 microscope (Zeiss, Jena, Germany) equipped with Colibri.2 LED illumination and an Apotome.2 system (Zeiss, Jena, Germany).

#### Actin/DAPI staining

Cell morphology was assessed by actin/DAPI staining. The samples were washed three times with PBS to remove the remaining culture medium, and the cells were fixed with paraformaldehyde (4%, v/v) for 30 min at room temperature. The cell membrane was permeabilized by incubating the samples in 0.1% Triton X-100-PBS solution for 5 min. The filamentous F-actin in the samples was stained with 1X Phalloidin iFluor 647 and nuclei were stained with a 1 μg/ml DAPI solution in PBS for 90 min at room temperature. The stained samples were observed using an Axio Observer. Z1 microscope (Zeiss, Jena, Germany) equipped with Colibri.2 LED illumination and an Apotome.2 system (Zeiss, Jena, Germany).

#### Confluence analysis

LIVE/DEAD micrographs were analyzed using ImageJ/Fiji software to assess cell confluence. The micrographs were transformed into a binary color scheme to facilitate the visualization of the cells occupying the surface. The confluence was calculated as the percentage of the pixels corresponding to cells with respect to the pixels of the total micrograph area.

#### Form factor analysis

We assessed the fibroblast morphology after culture in GelMA and in GelMA containing TuMV. To do this, cells were first stained with LIVE/DEAD reagents as described. We then measured the perimeter and area of randomly selected cells per micrograph (at least 20 cells per micrograph) using ImageJ/Fiji software. We calculated the form factor, as an indicator of cell morphology, using the formula 4π*(cell area/cell perimeter^2^). Values close to one correspond to round cells, while values that tend toward zero correspond to cells with an elongated and thin morphology.

### Biofabrication techniques

#### Chaotic 2D printing of GelMA-TuMV fibers

GelMA-TuMV fibers were fabricated as described previously (Frías-Sánchez et al., 2021). Briefly, GelMA was prepared in DPBS at a concentration of 10% (w/v) and 0.067% (w/v) LAP (CELLINK, Gothenburg, Sweden). Fibers were generated through chaotic 2D printing with a MiniJB device, following a protocol of a 270° counterclockwise rotation of the inner cylinder, followed by an 810° clockwise rotation of the reservoir, for one cycle. The diameter of the reservoir used during the printing process was 15 mm, and the diameter of the mixing shaft was 5 mm. The inner and outer cylinders were located at an eccentricity of 2.5 mm to generate the desired chaotic flows. Both cylinders were set at an angular velocity of 26.5 rpm. A bed of 400 µL glycerin was used as the supporting sacrificial ink for the deformation of the 1 µL drop of GelMA ink. After the printing process, the newly formed fibers were crosslinked with UV light at 365 nm for 15 s. The GelMA fibers were rinsed in PBS and placed in 24-well ultra-low-attachment (ULA) plates (Corning, United States). A coating suspension of TuMV at 10 μL/ml was added to the printed samples and incubated for 24 h at 4°C. Controls were covered in PBS. All samples were then transferred to a murine myoblast C2C12 cell suspension (4 × 10^4^ cells/mL) as well in 24-well ULA plates for a 3 h cell seeding step at 37°C in a humidified 5% CO_2_ atmosphere. After the seeding period, the cell suspension was replaced with fresh DMEM medium with 10% (v/v) FBS, and the samples were returned to the incubator.

#### TuMV hydrogel disks

GelMA disks of 1.6 mm diameter were manufactured by adding 25 µL droplets of 5% (w/v) GelMA to 96-well ULA plates. GelMA disks were later coated with 10 μg/ml of TuMV, EGF, or TuMV-EGF (conjugated) suspension and allowed to attach for 24 h at 4 °C. Subsequently, 4 × 10^4^ human BJ fibroblasts per well were seeded and cultured for 7 days.

#### 3D bioprinting

Bioinks were prepared by dissolving GelMA in PBS at a concentration of 5% (w/v) and adding 0.067% (w/v) LAP photoinitiator. TuMV or TuMV-Alexa555 were added to the GelMA solutions to a final concentration of 20 μg/ml. Prior to cell culture experiments, the hydrogel formulations were sterilized by filtration through 0.22 μm polyethersulfone (PES) filters. Bioinks were prepared by loading 1 × 10^6^ C2C12 cells/mL in the pristine GelMA or GelMA-TuMV hydrogels. The ink and bioink formulations were placed in 3 ml sterile cartridges and stored at 4°C overnight to generate a physical gel.

All printing experiments were performed using a BioX bioprinter from Cellink^®^ (Gothenburg, Sweden). The bioprinter cabinet was sterilized following the protocol recommended by the provider. The printing cartridges containing the inks or bioinks were attached to a sterile high-precision conical bioprinting nozzle (22G, 410 µm diameter) and mounted in the bioprinter. The print bed was cooled at 4°C. Straight lines of 14 mm were printed. We performed printings using three different pressure values (20, 30, and 40 KPa) and a constant printhead velocity (20 mm/s). The printed lines were photocrosslinked at 405 nm for 30 s.

The constructs were photographed using a USB microscope (Opti-TekScope, United States) to assess line thickness variation though image analysis using ImageJ/Fiji software. To evaluate the distribution of TuMV within the printed hydrogel, a line printed with GelMA loaded with TuMV-Alexa555 was cut with a new scalpel to obtain a cross section slide. The slide was observed using an Axio Observer. Z1 microscope (Zeiss, Jena, Germany) equipped with Colibri.2 LED illumination and an Apotome.2 system (Zeiss, Jena, Germany).

The constructs containing C2C12 cells were placed in DMEM culture medium and incubated at 37°C in a 5% CO_2_ atmosphere for 21 days. The constructs were stained at the endpoint using actin/DAPI and observed by microscopy as described above.

### Assessment of the rheological and mechanical properties of hydrogel samples

The rheology of the GelMA and GelMA-TuMV formulations was evaluated using a TA DHR-2 Discovery rheometer (TA Instruments, United Kingdom) equipped with a Peltier unit for temperature control. The parallel plate geometry (25 mm diameter) was chosen with a gap of 0.2 mm, and inks were tempered at 40°C prior to evaluation. Viscosities were measured as a function of temperature in a range from 10 to 40°C (cooling rate of 1°C/min) at a constant strain of 0.1%. The shear stress response was evaluated by varying the shear rate between 0.01 and 500 1/s in a rotational test at room temperature (∼22°C).

Cylinders (height: 4 mm, diameter: 14 mm) of GelMA and GelMA-TuMV hydrogels for use in compressive testing experiments were prepared by adding 1 ml of hydrogel to the wells of a 24-well plate and photopolymerizing at 405 nm for 30 s. Unconfined compression was conducted in a universal Testing System (UTS) with an Instron 3,365 dual column (Instron, Canton, MA) at room temperature and a compression rate of 200 μm min^−1^. The compression tests were replicated at least 3 times, and the mechanical properties of compression were obtained from the averaged compression curve.

### Statistical analysis

Data are presented as the mean ± SD from at least three repetitions (n = 3). Significant differences (*p* < 0.05) were found by analysis of variance (ANOVA) and Tukey post hoc tests.

## Data Availability

The raw data supporting the conclusion of this article will be made available by the authors, without undue reservation.
