# Supplementary material for: Gelatin-methacryloyl hydrogels containing turnip mosaic virus for fabrication of nanostructured materials for tissue engineering
Source: Front Bioeng Biotechnol. 2022 Sep 2;10:907601. doi: 10.3389/fbioe.2022.907601 (PMC9480610; doi:10.3389/fbioe.2022.907601)
Supplement: Supplementary file 1 [file DataSheet1.pdf]

Supplementary Material

Gelatin-methacryloyl (GelMA) hydrogels containing turnip mosaic virus (TuMV) for fabrication of nanostructured materials for tissue engineering

Ivonne González-Gamboa[1,3,#], Edith Velázquez-Lam[4,#], Matías José Lobo-Zegers[1,2,#], Ada Itzel Frías-Sánchez[1,2], Jorge Alfonso Tavares-Negrete[1,3], Andrea Monroy-Borrego[1,3], Jorge Luis Menchaca-Arrendondo[5], Laura Williams[6], Pablo Lunello[6], Fernando Ponz[4], Mario Moisés Alvarez[1,3,*], Grissel Trujillo-de Santiago[1,2,*]

[1] Centro de Biotecnología-FEMSA, Escuela de Ingeniería y Ciencias, Tecnológico de Monterrey, Monterrey, Nuevo León, México

[2] Departamento de Ingeniería Mecatrónica y Eléctrica, Escuela de Ingeniería y Ciencias, Tecnológico de Monterrey, Monterrey, Nuevo León, México

[3] Departamento de Bioingeniería, Escuela de Ingeniería y Ciencias, Tecnológico de Monterrey, Monterrey, Nuevo León, México

[4] Centro de Biotecnología y Genómica de Plantas, Universidad Politécnica de Madrid - Instituto Nacional de Investigación y Tecnología Agraria y Alimentaria (CBGP, UPM-INIA/CSIC), 28223 Madrid, Spain

[5] Centro de Investigación en Ciencias Físico Matemáticas (CICFIM). Facultad de Ciencias Físico-matemáticas. Universidad Autónoma de Nuevo León, México

[6] Agrenvec SL. Santiago Grisolia 2, 28760 Tres Cantos, Madrid, Spain

#Authors equally contributed to this work.

*Corresponding authors. Email: GTdS: grissel@tec.mx MMA: mario.alvarez@tec.mx

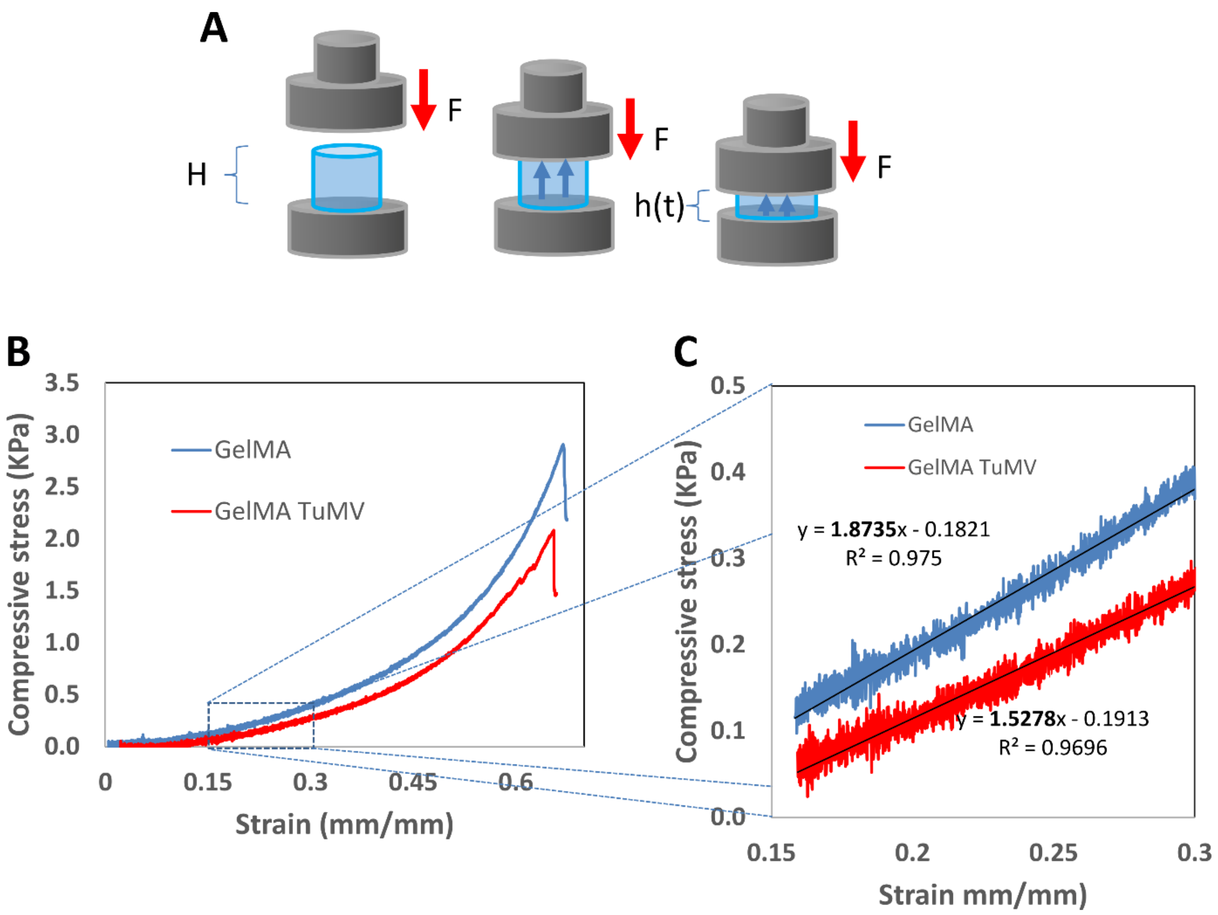

Figure S1. Compressive testing of GelMA and GelMA-TUMV hydrogel cylinders. (A) Schematic representation of the compressive testing experiments conducted in hydrogel cylinders casted in well plates. Cylinders 4 mm in height and 14 mm in diameter were prepared by adding 1 mL of hydrogel to the wells of a 24-well plate and photopolymerizing at 405 nm for 30 s. **(B)** Curves of average compressive stress versus strain for GelMA (blue symbols) and GelMA-TUMV (red symbols) cylinders; curves were constructed from the average of three independent compressive testing runs. **(C)** The compressive modulus (i.e., the slope of the straight-line section) of GelMA (blue symbols) and GelMA-TuMV (red symbols) hydrogels, as evaluated from the curves of compressive stress versus strain.

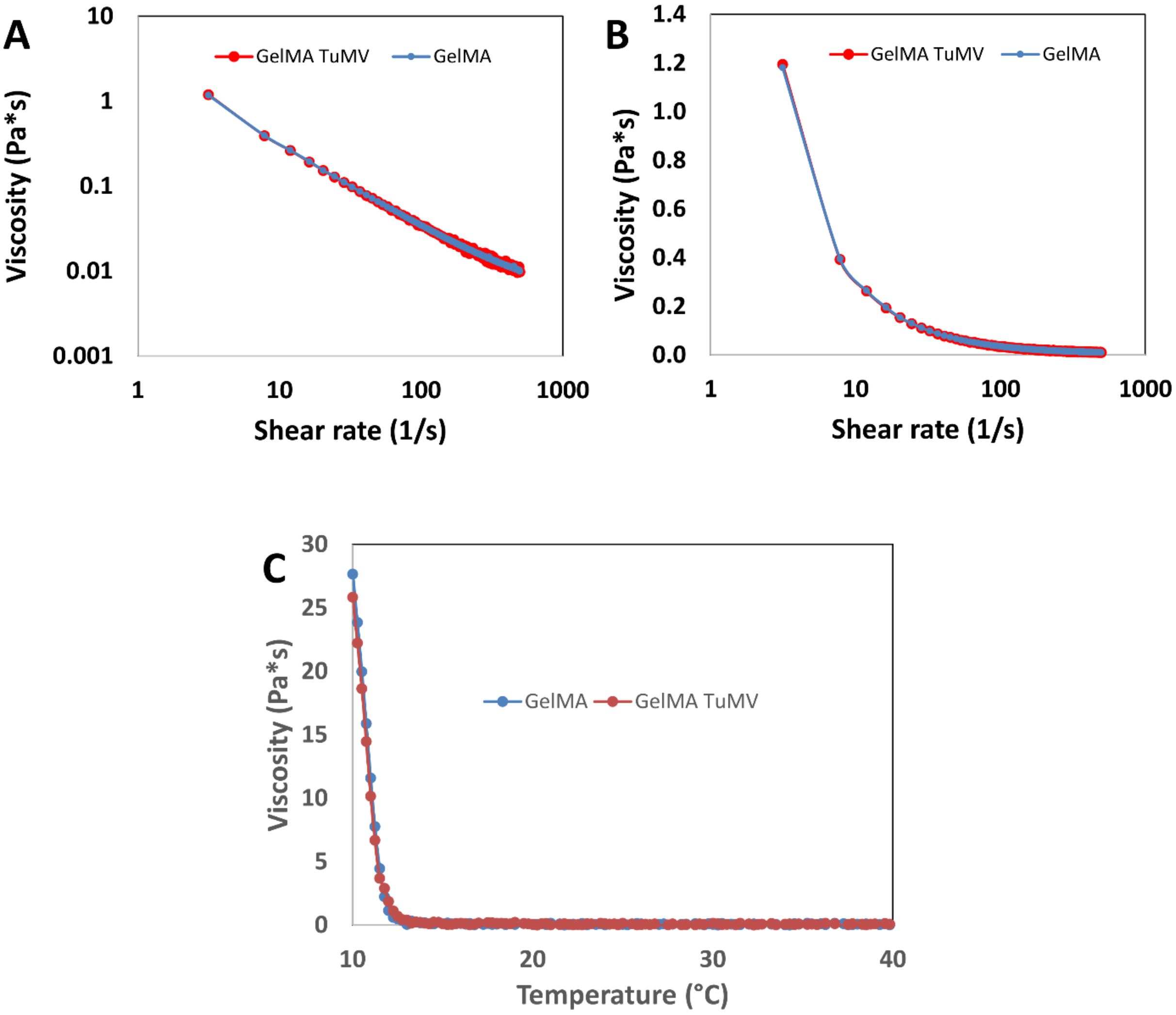

Figure S2. Rheological properties of GelMA and GelMA-TuMV hydrogels. (A-B) The viscosity versus shear rate curves of pristine GelMA (blue symbols) and GelMA-TuMV (red symbols) inks were similar. **(C)** The curves of viscosity of GelMA (blue symbols) and GelMA-TuMV (red symbols) inks at different temperatures were practically indistinguishable.
